# The lncRNA MALAT1/miR-30/Spastin Axis Regulates Hippocampal Neurite Outgrowth

**DOI:** 10.3389/fncel.2020.555747

**Published:** 2020-10-20

**Authors:** Tao Jiang, Zhenbin Cai, Zhisheng Ji, Jianyu Zou, Zhi Liang, Guowei Zhang, Yaozhong Liang, Hongsheng Lin, Minghui Tan

**Affiliations:** ^1^Department of Orthopaedics, The First Affiliated Hospital of Jinan University, Guangzhou, China; ^2^Department of Orthopaedics, Guangzhou Eighth People’s Hospital, Guangzhou Medical University, Guangzhou, China

**Keywords:** neurite outgrowth, spastin, miR-30, MALAT1, microtubule severing

## Abstract

Spastin, a microtubule-severing enzyme, is important for neurite outgrowth. However, the mechanisms underlying the post-transcriptional regulation of spastin during microtubule-related processes are largely unknown. We demonstrated that the spastin expression level is controlled by a long non-coding RNA (lncRNA) metastasis-associated lung adenocarcinoma transcript 1 (MALAT1)/microRNA-30 (miR-30) axis during neurite outgrowth. The miR-30 expression level decreased in hippocampal neurons with increasing days in culture, and miR-30 overexpression suppressed while miR-30 inhibition promoted neurite outgrowth in hippocampal neurons. Spastin was validated as a target gene of miR-30 using the luciferase reporter assay. The protein expression, microtubule severing activity, and neurite promoting effect of spastin were suppressed by the overexpression of miR-30 mimics and increased by miR-30 inhibitors. MALAT1 expression increased during neurite outgrowth and MALAT1 silencing impaired neurite outgrowth. miR-30 was a sponge target of MALAT1 and MALAT1/miR-30 altered neurite outgrowth in hippocampal neurons. MALAT1 overexpression reversed the inhibitory effect of miR-30 on the activity of a luciferase reporter construct containing spastin, as well as spastin mRNA and protein expression, indicating that spastin was a downstream effector of MALAT1/miR-30. The MALAT1/miR-30 cascade also modulated spastin-induced microtubule severing, and the MALAT1/miR-30/spastin axis regulated neurite outgrowth in hippocampal neurons. This study suggests a new mechanism governing neurite outgrowth in hippocampal neurons involving MALAT1/miR-30-regulated spastin expression.

## Introduction

Spastin, a microtubule-cutting protein in the “ATPases associated with diverse cellular activities” (AAA) protein family (Sharp and Ross, 2012), was first discovered in hereditary spastic paraplegia (HSP; Hazan et al., 1999), a neurodegenerative disease involving the degeneration of axons in the bilateral corticospinal tract of the spinal cord (McDermott et al., 2000). Spastin is involved in neuronal development, especially the formation of neurite branches, because of its microtubule-cutting properties (Yu et al., 2008; Riano et al., 2009; Brill et al., 2016). Silencing spastin changes microtubule stability, curls axons, and impairs anterograde and retrograde axonal transport in human HSP patients (Kasher et al., 2009), zebrafish (Wood et al., 2006), and cultured neurons (Yu et al., 2008; Korulu and Karabay, 2011; Fassier et al., 2013). Spastin regulates a variety of neuronal functions *via* its interactions with other proteins, such as Atlastin, which affects ER re-localization during axon regeneration (Sanderson et al., 2006; Rao et al., 2016), NA14, which promotes the co-fractionation of spastin with gamma-tubulin in the centrosome (Errico et al., 2004), ESCRT-III and CHMP1B complex, which coordinates intracellular membrane traffic, mitotic spindle disassembly, and nuclear envelop sealing, and protrudin, which facilitates axon formation (Zhang et al., 2012). We previously showed that spastin interacts with collapsing response-mediator protein-5 (CRMP5) to regulate neurite outgrowth (Ji et al., 2018). These reports suggest an important role in protein-protein interactions in the activity regulation of spastin during neuronal development. The post-transcriptional regulation of spastin is also critical for spastin expression but the underlying mechanisms remain unknown.

MicroRNAs (miRNAs) are enriched in the central nervous system and are directly involved in the regulation of gene expression. miRNAs regulate neuronal development and process regeneration in many ways (Rajman and Schratt, 2017). For instance, miR-29a promotes axonal branching (Li et al., 2014), miR-134 increases dendritic branching (Gaughwin et al., 2011), miR-138 inhibits dendritic spine maturation (Siegel et al., 2009), miR-132 promotes dendritic spine maturation (Yang et al., 2012), and miR-101 regulates presynaptic and post-synaptic development (Lippi et al., 2016). Thus, miRNAs are involved in every phase of neuron development including neurite formation, extension, and maturity. miR-33a has been identified as a new target for the treatment of HSP caused by mutations in the spastin gene (Nakazeki et al., 2019), and miR-96 and miR-182 are also involved in the post-transcriptional regulation of spastin protein levels (Henson et al., 2012). For these reasons, the post-transcriptional regulation of spastin expression has become an important focus of research.

Long non-coding RNA (lncRNA) also plays an important role in the transcription and post-transcriptional regulation of gene expression, especially in the regulation of the expression of miRNAs *via* the decoy or sponge effect (competitive inhibition; Salmena et al., 2011; Li et al., 2018a). Forty percentage of lncRNA is expressed in the brain (Derrien et al., 2012), and it has been identified as a new regulator of neuronal differentiation and development. lncRNA MAP2k4 promotes neuronal proliferation and inhibits neuronal apoptosis through the miR-199a/FGF1 pathway (Lv, 2017). The inhibition of endogenous lncRNA IGF2AS promotes neuronal growth (Zhang et al., 2016), and the knockdown of lncRNA Pnky promotes neuronal differentiation (Ramos et al., 2015). lncRNA metastasis-associated lung adenocarcinoma transcript 1 (MALAT1) also plays a key role in neuronal growth and development (Zhang et al., 2016). However, how miRNAs and MALAT1 regulate spastin expression in hippocampal neurons has not yet been reported.

The present study shows that lncRNA MALAT1 promotes the neurite outgrowth of hippocampal neurons by counteracting the negative effect of miR-30 on spastin expression and microtubule severing activity.

## Materials and Methods

### Cell Culture and Transfection

Primary hippocampal neurons were cultured as previously described (Tan et al., 2015). All animal procedures were carried out following the Guide for the Care and Use of Laboratory Animals from the NIH and were approved by the Jinan University Institutional Animal Care and Use Committee. Briefly, hippocampi from 1-day-old Sprague–Dawley rat pups were dissected and incubated with trypsin (0.125%) at 37°C for 15 min. The hippocampi were washed with Dulbecco’s Modified Eagle Medium (DMEM)/F12 containing 10% fetal bovine serum (Gibco) to stop the trypsin activity. The cells were plated on glass coverslips that were previously coated with poly-L-lysine (Sigma–Aldrich, USA) at a density of 1 × 10^4^ cells/cm^2^. After the cells were attached, the medium was replaced by neurobasal supplemented with 2% B27 (Gibco). Cultures were maintained at 37°C in a humidified 5% CO_2_ atmosphere. Plasmids were transfected at 3 days *in vitro* (DIV3) by the calcium phosphate method and were then cultured with full media for another 2 days (DIV5). Hippocampal neurons were subjected to immunofluorescence and imaged using a confocal microscope (Zeiss, Germany).

HT22 and COS1 cells were obtained from the Chinese Academy of Sciences Cell Bank (Shanghai, China), cultured in DMEM medium (Gibco, USA) with 10% fetal bovine serum, and maintained in a humidified incubator at 37°C with 5% CO_2_. The spastin (NCBI Reference Sequence: NM_001108702.2) cDNA was subcloned into the pEGFP-C1 (Clontech, Mountain View, CA, USA) as reported (Ji et al., 2018). Fragments (NC/miR-30 mimic/inhibitor) or plasmids were performed using lipofectamine 2000 (Invitrogen, Carlsbad, CA, USA) according to the manufacturer’s instructions.

### Luciferase Reporter Assay

**Figure 1:**
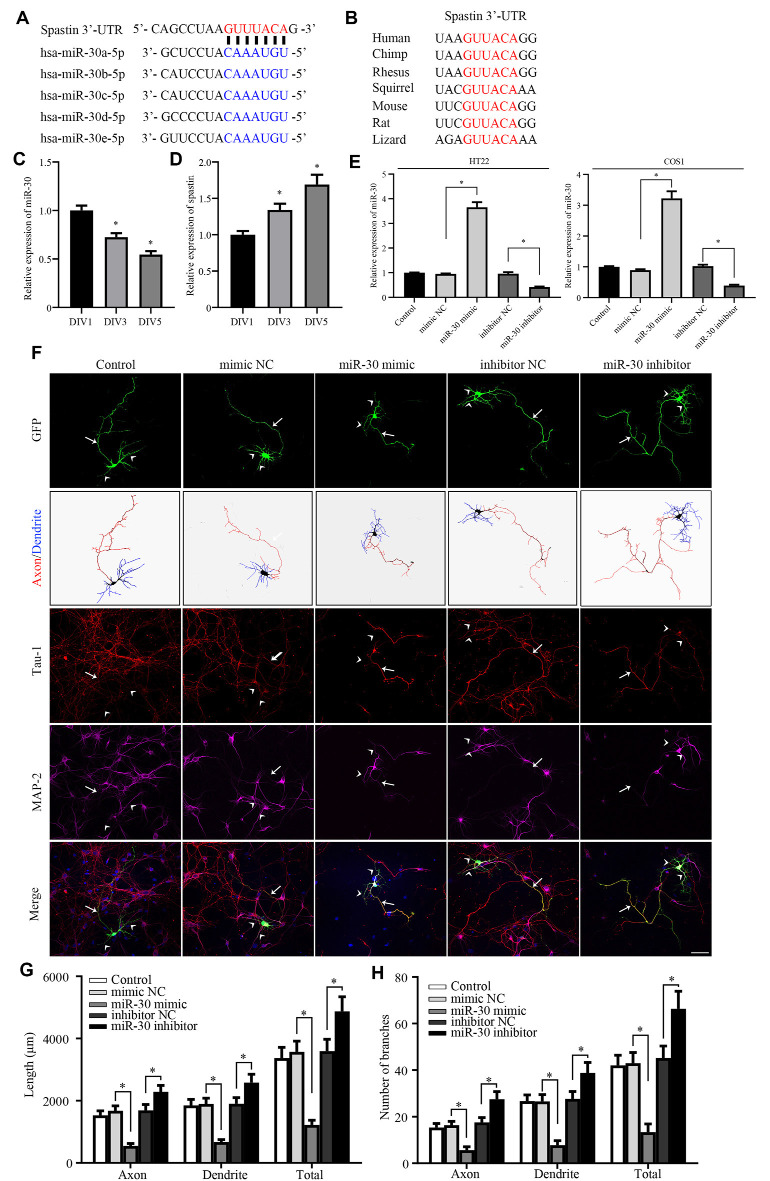
miR-30 inhibits neurite outgrowth in cultured hippocampal neurons. **(A)** Bioinformatic analysis using the TargetScan tool showing the sequence of the spastin 3′-UTR is targeted by five members of the miR-30 family. **(B)** Alignment of the miR-30-targeted spastin 3′-UTR sequences from different species shows high homology. The relative expression levels of miR-30 **(C)**, and spastin **(D)** obtained using qPCR in DIV1, DIV3, and DIV5 stages of hippocampal neuron development (*n* = 3). **(E)** HT22 and COS1 cells were cultured and transfected with miR-30 mimics and inhibitor fragments. qPCR was performed to determine the level of miR-30 expression. The miR-30 mimic or inhibitor fragments together with the GFP plasmid were then transfected into DIV3 hippocampal neurons for 48 h and subjected to immunofluorescence with GFP, MAP2 (for dendrite), and Tau-1 (for axon) antibodies to determine neuronal morphology. Nuclei were stained with DAPI. The tailed arrow indicates the axon, while arrows with no tail indicate dendrites. Panel **(F)** shows representative images. The length **(G)** and the number of branches **(H)** of neurites were also measured (*n* = 4; neuron number >30/group of each experimental repeat). *P < 0.05. Scale bar, 50 μm.

A luciferase reporter vector pGL3-Control vector (Promega, USA) was used to generate the luciferase reporter construct. The spastin (wild type, WT or mutant, MUT) 3′ untranslated region (UTR; partial, 200 nt) with the binding site of miR-30 was cloned into the pGL3-Control vector. The sequences of spastin and MALAT1 are shown in Figures 1, 5. The luciferase activity assays were performed using the Dual-Luciferase Assay System (Promega, USA) as described previously (Wang et al., 2005), with simultaneous detection of an internal reporter a Renilla luciferase plasmid to minimize differences in cell viability and transfection efficiency. The HT22 and COS1 cells were plated for transfection. Lipofectamine 2000 (Invitrogen, Carlsbad, CA, USA) was used to transfect the constructed WT or MUT reporters. Then, 48 h after transfection, the samples were collected and tested according to the instructions of the Dual-Luciferase Reporter Assay System. Fragments of miR-30 mimics and inhibitors and negative controls were purchased from Genepharma (Shanghai, China). The sequences are: (1) miR-30 mimic sense, 5′-UGUAAACAUCCUCGACUGGAAG-3′; anti-sense, 5′-UCCAGUCGAGGAUGUUUACAUU-3′; (2) miR-30 inhibitor, 5′- CUUCCAGUCGAGGAUGUUUACA-3′.

**Figure 2:**
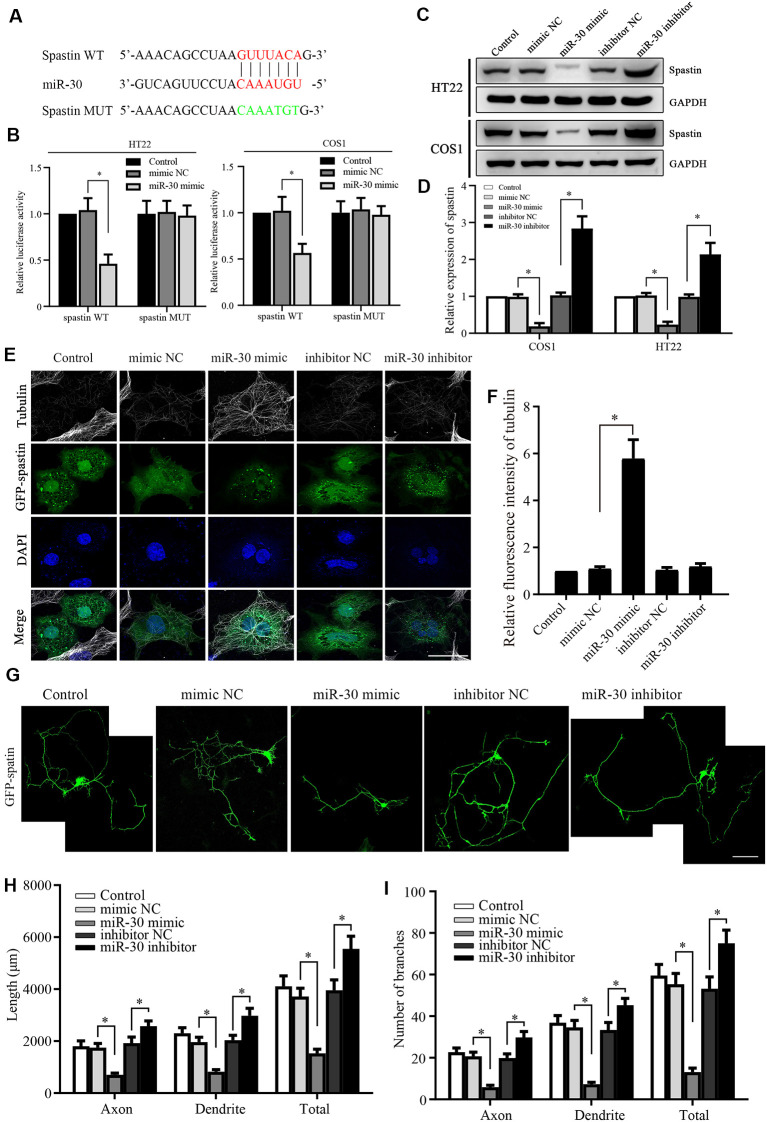
Spastin is targeted by miR-30 to regulate microtubule dynamics and neurite outgrowth.** (A)** Luciferase reporter plasmids of spastin WT and MUT were constructed to determine the effect of miR-30. The targeted and mutant sequences are shown. **(B)** Spastin luciferase reporter plasmids were co-transfected with miR-30 mimic or control. The luciferase activity was measured in HT22 and COS1 cells (*n* = 3). **(C)** Protein expression levels of spastin were determined by western blotting of extracts from HT22 and COS1 cells transfected with miR-30 mimics or controls (*n* = 3) and relative expression levels of spastin vs. GAPDH were quantified **(D)**. **(E)** The mimic or inhibitor of miR-30 was co-transfected together with the GFP-spastin plasmid into COS1 cells, which were then subjected to immunofluorescent antibody staining for tubulin (white) and GFP (green). Nuclei are stained with DAPI (blue); Scale bar, 25 μm. **(F)** The relative fluorescence intensities of tubulin in COS1 cells were quantified (*n* = 3; cell number >50/group of each repeat). **(G)** The mimics or inhibitor of miR-30 were co-transfected with the GFP-spastin plasmid into DIV3 hippocampal neurons for 48 h, and the neurons were immunostained to assess morphology; Scale bar, 50 μm. The lengths **(H)** and the number of branches **(I)** of neurites were measured (*n* = 3; neuron number >30/group of each experimental repeat). *P < 0.05.

**Figure 3:**
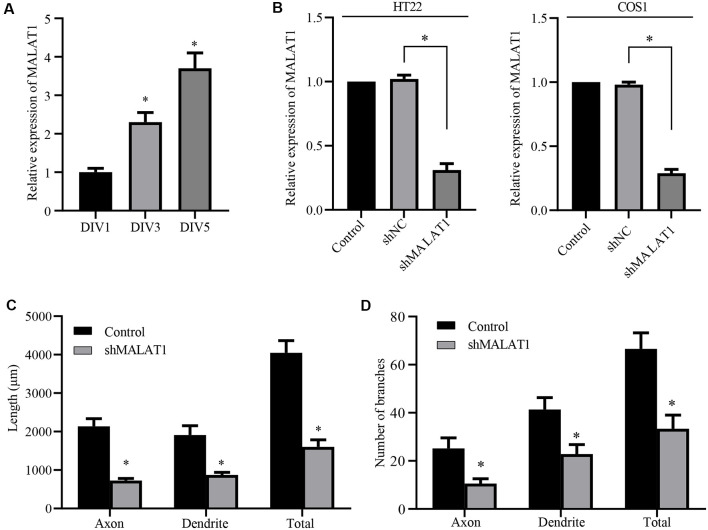
Long non-coding RNA (lncRNA) MALAT1 is critical for neurite outgrowth of hippocampal neurons.** (A)** qPCR data shows that the levels of lncRNA MALAT1 increased during hippocampal neuronal development. **(B)** Plasmids targeting MALAT1 (shMALAT1) were transfected into the HT22 and COS1 cells, and the expression levels were determined by qPCR. DIV3 hippocampal neurons were transfected with shMALAT1 plasmids, and the lengths **(C)** and the number of branches **(D)** were counted. *P < 0.05; *n* = 3.

**Figure 4:**
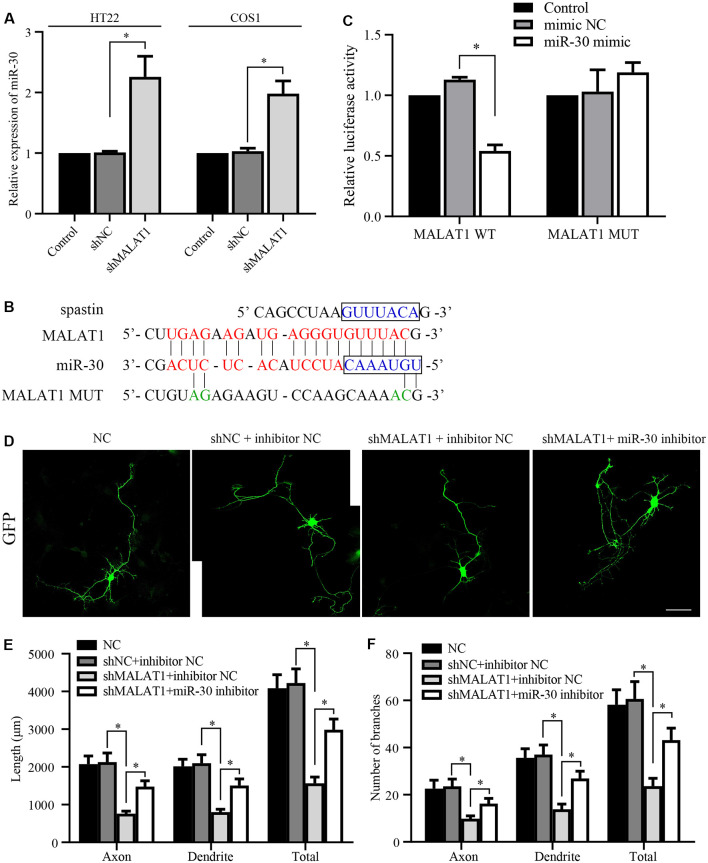
lncRNA MALAT1 targets miR-30 during neural development. **(A)** The expression levels of miR-30 were determined by qPCR in HT22 and COS1 cells transfected with shMALAT1 or control plasmids. **(B)** Alignments of the luciferase reporter plasmids encoding MALAT1 WT or MUT against miR-30. **(C)** The reporter plasmids were transfected together with or without miR-30 mimics, and the relative luciferase activity was quantified. **(D)** Representative images of the cultured neurons that were transfected with the shMALAT1 plasmids or miR-30 inhibitors. Neurite lengths **(E)** and the number of branches **(F)** were quantified (neuron number >30/group of each experimental repeat). *P < 0.05; *n* = 3; Scale bar, 50 μm.

**Figure 5:**
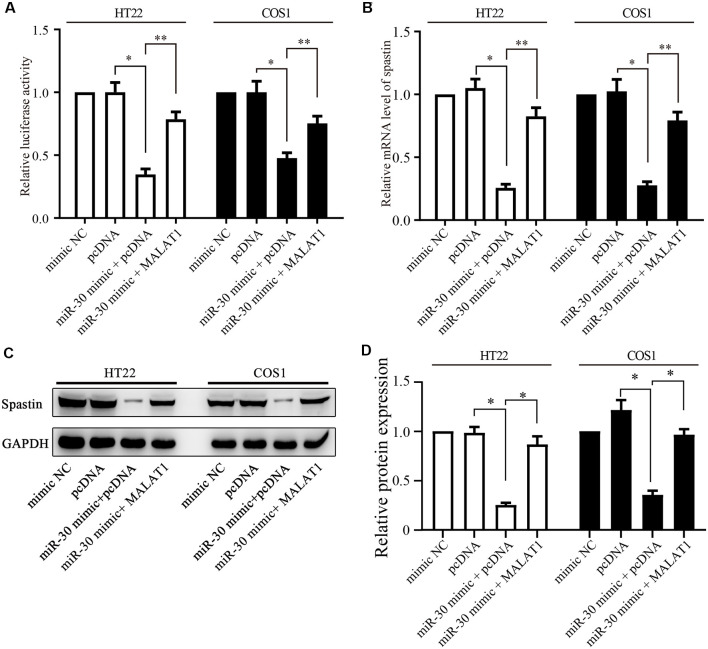
MALAT1 regulates spastin expression through miR-30. **(A)** HT22 and COS1 cells were co-transfected with the spastin luciferase reporter plasmid together with miR-30 or with miR-30 and MALAT1; or with controls. The relative luciferase activity is shown. **(B)** mRNA and protein expression **(C)** of spastin was determined by qPCR and western blotting in HT22 and COS1 cells transfected with the indicated plasmids or mimics. **(D)** Relative protein expression of spastin vs. GAPDH from **(C)** was quantified. *P < 0.05, **P < 0.01; *n* = 3.

### Quantitative Real-Time PCR

TRIzol reagent (Invitrogen) was applied to total RNA extracts of HT22 or COS1 cells according to the manufacturer’s instructions. An EasyScript cDNA Synthesis SuperMix (TransGen Biotech, China) was used to reverse transcribe the mRNA. A TransScript^®^ miRNA First-Strand cDNA Synthesis SuperMix (TransGen Biotech, China) was used for miRNA cDNA synthesis. The quantitative reverse transcription-polymerase chain reaction (PCR) was performed using TransStart Top Green quantitative PCR (qPCR) SuperMix (TransGen Biotech, China). miR-30 and mRNA expression levels were normalized to those of U6 and glyceraldehyde 3-phosphate dehydrogenase (GAPDH) using a delta-delta-Ct method as described previously (Livak and Schmittgen, 2001). All PCR reactions were repeated at least three times. The primers specific to mature miR-30 were purchased from Qiagen. The following sense and anti-sense primers were used:

spastin (HT22): 5′-GAACCCGTCTTCTTTCTCGTC-3′, 5′-AATGGAGATGTACTCGAAGGC-3′;

spastin (COS1): 5′-AGCGGAACCTGTACTATTTCTC-3′, 5′-CTGCTTTCTCATCCTCATCGAT-3′;

MALAT1: 5′-CTCACTAAAGGCACCGAAGG-3′, 5′-GGCAGAGAAGTTGCTTGTGG-3′.

### Western Blotting

Western blotting was performed as previously described (Cai et al., 2019). Samples were harvested and lysed using a radioimmunoprecipitation assay (RIPA) lysis buffer containing protease inhibitors (Beyotime, China). Protein concentrations in the samples were determined using a BCA protein assay kit (Beyotime, China). Proteins were separated on a 10% sodium dodecyl sulfate-polyacrylamide gel (SDS–PAGE) and transferred to a polyvinylidene fluoride (PVDF) membrane (Millipore, USA). The membrane was blocked with 5% non-fat milk for 1 h and incubated with primary antibodies against spastin (Santa Cruz, #sc-374068), GAPDH (Abcam, Cambridge, UK; #ab8245) overnight at 4°C. After secondary antibody (Abclonal Biotechnology, China) incubation for 1 h at room temperature, proteins on the membrane were detected using enhanced chemiluminescence (ECL; Beyotime, China).

### Immunofluorescence

Immunofluorescence was performed as previously described (Tan et al., 2013; Jiang et al., 2020). Samples were fixed in 4% paraformaldehyde (Sigma–Aldrich, USA) for 30 min and then were permeabilized in phosphate-buffered saline (PBS) containing 0.1% Triton X-100. After blocking with 3% corresponded serum, the cells were incubated with primary antibodies (GFP, Abcam, #ab290, 1:1,000; Tubulin, Abcam, #ab6160, 1:1,000) overnight at 4°C, and then labeled with appropriate fluorescent-tagged secondary antibodies, Alexa Fluor 488 (Thermo Fisher Scientific), Alexa Fluor 555 (Thermo Fisher Scientific) and Alexa Fluor 647 (Thermo Fisher Scientific). The following primary antibodies were used: Tubulin (Abcam, #ab6160) and green fluorescent protein (GFP; Abcam, #ab290). The cells were mounted in Fluro-Gel II containing 4′,6-diamidino-2-phenylindole (DAPI; Electron Microscopy Sciences, Hatfield, PA, USA) and imaged with a Carl Zeiss LSM 780 confocal microscope (Zeiss, Germany).

### Neuronal Morphometry

Hippocampal neuron morphometry was performed as previously reported (Tan et al., 2013; Jiang et al., 2020). Generally, transfected neurons were immune-stained with antibodies against GFP (Abcam) to reveal overall cell morphology; MAP2 to identify dendrites; Chemicon, Temecula, CA, USA, and Tau-1 (Chemicon) to identify axons; Images were captured through an LSM 780 confocal microscope (Carl Zeiss, Germany) in a blinded manner. At least 30 neurons from each experimental group were used for the qualification of neurite/dendrites/axons using Image-Pro Plus 6 software (Media Cybernetics, Silver Spring, MD, USA).

### Statistical Analysis

The experimental data are expressed as mean ± standard error of the mean (SEM). GraphPad Prism 5 software was used to make graphs and perform statistical analyses. All data were tested for normality using the Shapiro–Wilk *W* method of analysis. Analyses were performed using a *t*-test (for single comparisons) or one-way analysis of variance (ANOVA, for multiple comparisons). *P* < 0.05 was considered statistically significant.

## Results

### miR-30 Is Involved in Neurite Development

Our previous report showed that spastin regulates neurite outgrowth by modulating cytoskeleton dynamics (Ji et al., 2018). Because the detailed mechanism of post-transcriptional regulation of spastin remains largely unknown, we tested whether miRNAs targeted spastin expression. TargetScan (Agarwal et al., 2015), miRDB (Wang, 2016), and picTar (Krek et al., 2005) online tools were used to conduct bioinformatics analyses, which showed that all members of the miR-30 subfamily (from miR-30a to miR-30e) could potentially influence spastin expression (Figure 1A) *via* the same sequence. The 3′-UTR of spastin that matched the miR-30 sequence is highly conserved among multiple species (Figure 1B), suggesting that miR-30 might affect the expression of spastin; however, the role of miR-30 in neurite outgrowth is unknown. We therefore first tested whether miR-30 is involved in neurite development. Analysis of the expression levels of miR-30 and spastin revealed that miR-30 and spastin expression were inversely correlated; miR-30 (Figure 1C) expression decreased while spastin expression (Figure 1D) increased in hippocampal neurons with increasing days in culture. A mimic and an inhibitor of miR-30 were synthesized and validated in HT22 and COS1 cells because these cells had high transfection efficiency (Figure 1E), and then transfected into cultured hippocampal neurons. As shown in Figure 1F, miR-30 mimics significantly suppressed while miR-30 inhibitors promoted neurite outgrowth. Neurite lengths (Figure 1G) and the number of branches (Figure 1H) of axons (stained with Tau-1 antibody), dendrites (stained with MAP2 antibody), and the total were measured. These data suggest that miR-30 expression is negatively regulated and critically important for neurite outgrowth of hippocampal neurons.

### miR-30 Targets Spastin to Regulate Microtubule Dynamic Activity and Neurite Outgrowth

To confirm that miR-30 directly targets spastin, we constructed luciferase reporter plasmids encoding the 3′-UTR of spastin WT and MUT (Figure 2A). After the transfection of the reporters with or without miR-30 mimics, luciferase activity was measured. miR-30 mimics suppressed the activity of the WT spastin, but not that of the MUT reporter, in both HT22 and COS1 cells (Figure 2B). Western blotting showed that miR-30 mimics reduced while the inhibitors increased spastin expression in both cell lines (Figures 2C,D). These data indicate that miR-30 decreases spastin expression during the development of hippocampal neurons.

Next, we tested whether the miR-30 regulation of spastin expression affected spastin-dependent microtubule severing and neurite outgrowth. To visualize microtubule severing activity, GFP-spastin encoding plasmids were co-transfected into COS1 cells with or without miR-30 mimics or inhibitors. COS1 cells are very flat and hence permit more spatial resolution than is possible with neurons. The GFP-spastin plasmid significantly induced microtubule severing (Figure 2E, control group). miR-30 mimics inhibited severing, leading to increased microtubule staining (Figure 2E, miR-30 mimic group), whereas miR-30 inhibitors did not affect (Figure 2E). Quantification of the relative fluorescence intensities of the microtubules (Figure 2F) showed that miR-30 mimic transfection affected spastin-promoted microtubule severing. Similar co-transfections in cultured hippocampal neurons showed that GFP-spastin-induced neurite outgrowth (Ji et al., 2018) could be inhibited by transfection with miR-30 mimics (Figure 2G), whereas neurite outgrowth was increased further by transfection by miR-30 inhibitors. Figure 2H shows the lengths of axons, dendrites, and total neurites, and Figure 2I shows the number of branches. Taken together, these data indicate that the spastin is regulated by miR-30 and that this affects microtubule severing and neurite outgrowth of hippocampal neurons.

### lncRNA MALAT1 Is Involved in Neurite Outgrowth of Hippocampal Neurons

Although miR-30 is a known target of lncRNA MALAT1 (Yi et al., 2019), the role of MALAT1 in neurite outgrowth of primary cultured neurons and the detailed mechanism underlying the effect of the MALAT1/miR-30 signaling on spastin expression remain largely unknown. Thus, we first determined whether MALAT1 was involved in neurite outgrowth of hippocampal neurons. Analysis of MALAT1 expression levels in cultured hippocampal neurons at DIV1, DIV3, and DIV5 demonstrated that MALAT1 expression increased with increasing days in culture (Figure 3A), indicating a positive correlation between MALAT1 expression and the development of hippocampal neurons. To verify the role of MALAT1 in hippocampal neurons, a MALAT1 shRNA plasmid (shMALAT1) was constructed, and its efficiency in reducing MALAT1 expression was verified by qPCR in HT22 and COS1 cells. As shown in Figure 3B, transfection with shMALAT1 plasmids significantly suppressed the expression of MALAT1. When shMALT1 was overexpressed in cultured hippocampal neurons, it significantly suppressed outgrowth, as shown by the decrease in the length (Figure 3C) and branch number (Figure 3D) of axons, dendrites, and total neurites. These data suggest that MALAT1 is important for neural outgrowth in hippocampal neurons.

### MALAT1 Regulates Neurite Outgrowth Through miR-30

Next, we tested whether MALAT1 mediates neurite outgrowth *via* an effect on miR-30 expression. After transfection with the shMALAT1 plasmid, the expression level of miR-30 was determined by qPCR. As shown in Figure 4A, MALAT1 silencing significantly upregulated miR-30 levels in both HT22 and COS1 cell lines. The MALAT1 sponge targeting sequence against miR-30 is shown in Figure 4B. The sequence in the spastin 3′-UTR aligned with the miR-30 targeted MALAT1 sequence (Figure 4B, blue color), suggesting a potential competing relationship between MALAT1 and spastin concerning miR-30. To confirm the relationship between MALAT1 and miR-30, MALAT1 WT and MUT reporters were separately transfected into COS1 cells (Figure 4C). Overexpression of miR-30 mimics suppressed the luciferase reporter activity of MALAT1 WT, but not that of MUT, suggesting a direct regulatory interaction between MALAT1 and miR-30. To clarify whether MALAT1 regulates the growth of protrusions in hippocampal neurons through miR-30, hippocampal neurons were co-transfected with shMALAT1 plasmids with or without miR-30 inhibitors. ShMALAT1 significantly and consistently reduced neurite outgrowth (Figure 4D), and overexpression of miR-30 inhibitors rescued the reduction in neurite outgrowth induced by shMALAT1 transfection. Neurite length (Figure 4E) and the number of branches (Figure 4F) were also quantified. These data indicate that MALAT1 regulates neurite outgrowth by suppressing miR-30 expression.

### MALAT1 Regulates the Expression of Spastin Through miR-30

Next, we determined whether MALAT1 regulates spastin expression *via* miR-30. A luciferase reporter of spastin WT was transfected together with miR-30 mimics or MALAT1 into HT22 and COS1 cells. Luciferase activity assays revealed that overexpression of miR-30 mimics significantly reduced reporter activity (Figure 5A). Overexpression of MALAT1 alleviated the negative effect of miR-30 mimics on spastin reporter activity (Figure 5A). These results were confirmed by qPCR measurements of spastin mRNA levels. The miR-30 mimic reduced the mRNA level of spastin, but this effect was reversed by MALAT1 overexpression (Figure 5B). Additionally, western blotting showed that suppression of spastin expression by overexpression of miR-30 mimics was rescued by MALAT1 overexpression (Figures 5C,D). These data indicate that MALAT1 regulates the expression of spastin through the inhibition of miR-30.

### The MALAT1/miR-30/Spastin Axis Regulates Microtubule Severing and Neurite Outgrowth

**Figure 6:**
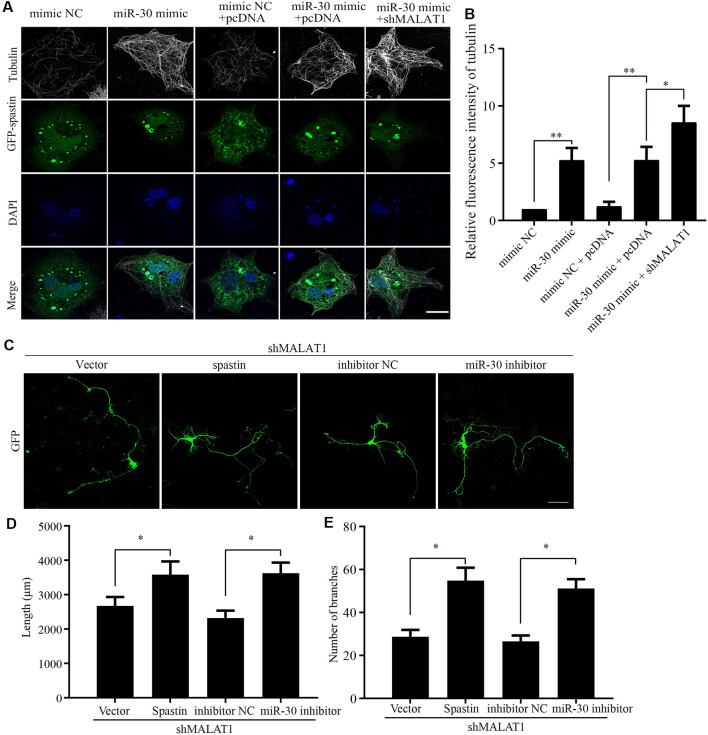
MALAT1/miR-30/spastin signaling axis regulates microtubule severing and neurite outgrowth.** (A)** COS1 cells were transfected with the GFP-spastin plasmid together with the miR-30 mimic, the miR-30 mimic plus shMALAT1, or controls. The cells were then subjected to immunofluorescent antibody staining for tubulin (white) and GFP (green). Nuclei are stained with DAPI (blue). Representative images are shown; Scale bar, 25 μm. **(B)** The relative fluorescence intensities of tubulin in **(A)** were quantified (*n* = 3; cell number > 50/group of each repeat). *P < 0.05, **P < 0.01. The cultured neurons were co-transfected with the shMALAT1 plasmid together with spastin encoding plasmids or the miR-30 inhibitor fragments. Then, the neurons were subjected to immunofluorescence for morphological analysis. Representative images are shown in **(C)**. Neurite lengths **(D)** and the number of branches **(E)** were also measured (*n* = 3; neuron number >30/group for each repeat). *P < 0.05; Scale bar, 50 μm.

Because the previous experiments demonstrated that the actions of MALAT1, miR-30, and spastin were intimately related, we next sought to confirm the effect of MALAT1/miR-30/spastin signaling on microtubule severing. COS1 cells were co-transfected with a GFP-spastin plasmid together with or without miR-30 mimics or shMALAT1 encoding plasmids. As shown in Figure 6A, the microtubule severing induced by GFP-spastin expression was inhibited by miR-30 mimics, and further promoted by shMALAT1. Tubulin fluorescence intensity was quantified and the data are shown in Figure 6B. To confirm the effect of MALAT1/miR-30/spastin axis on neurite outgrowth, hippocampal neurons were co-transfected with shMALAT1 plasmids, with or without spastin or miR-30 inhibitor. As shown in Figure 6C, overexpression of spastin or the miR-30 inhibitor promoted neuron development, rescuing the inhibitory effect of MALAT1 silencing. The neurite length (Figure 6D) and the number of branches (Figure 6E) were quantified. These data indicate that the MALAT1/miR-30/spastin axis controls microtubule dynamics and neurite outgrowth.

## Discussion

In this study, we provide new information that clarifies how spastin expression is regulated during neurite outgrowth. Bioinformatic analysis and luciferase reporter assays showed that spastin expression was directly targeted and inhibited by miR-30. miR-30 also decreased microtubule severing and neurite outgrowth by negatively regulating spastin expression. Moreover, lncRNA MALAT1 was important for neurite outgrowth, and MALAT1 sponged miR-30. MALAT1 positively regulated microtubule severing and neurite outgrowth *via* miR-30/spastin signals. Taken together, the results point to the existence of a novel MALAT1/miR-30/spastin axis for the regulation of cytoskeleton dynamics and neurite outgrowth in cultured hippocampal neurons.

Spastin is abundantly expressed in developing neural tissues and is mainly distributed at the branches of axons and distal regions of axons (Claudiani et al., 2005). Activated spastin is usually a circular hexamer that binds and anchors microtubules through the microtubule-binding domain (MBD) region and severs microtubules through the AAA domain (Salinas et al., 2005; Solowska and Baas, 2015). Intensive investigations have revealed that protein-protein interactions are important for the regulation of spastin expression; however, the post-transcriptional regulation of spastin has been less investigated. Previous reports have shown that spastin is regulated by miR-96, miR-182, and miR-367 (Henson et al., 2012), but the function of these miRNAs in neuronal development was not addressed. SPAST was identified as a target of miR-33 and a potential therapeutic target for the treatment of atherosclerosis (Nakazeki et al., 2019). Inhibition of miR-33a *via* locked nucleic acid (LNA)-anti-miR ameliorates the pathological phenotype of HSP-SPG4 patients induced pluripotent stem cell-derived cortical neurons (Nakazeki et al., 2019). Here, we found that miR-30 targets spastin to regulate hippocampal neurite outgrowth. Our findings, in addition to elucidating the mechanism of post-transcriptional regulation of spastin, open the prospect of developing novel potential therapeutic targets for the treatment of HSP caused by mutations in spastin.

Less than 2% of RNA in the human genome can be translated into proteins, and the remaining RNA is collectively referred to as non-coding RNA (including short-chain miRNA, medium-length snoRNA, and long-chain lncRNA; Taft et al., 2010). miRNAs are small non-coding RNA molecule of 20–25 nucleotides that regulates gene expression through translation inhibition or mRNA degradation, mainly through their interactions with the 3′UTRs of target genes (Egawa et al., 2016; Salas-Huetos et al., 2019). The miR-30 family consists of five members, including miR-30a, miR-30b, miR-30c, miR-30d, and miR-30e. Members of the miR-30 family share homologous sequences with spastin, and the seed sequences are identical (Figure 1). miR-30 family members have been implicated in regulating a variety of biological processes, such as tumor development and progression (Mao et al., 2018), islet cell development (Ozcan, 2009), adipogenesis (Zaragosi et al., 2011), and osteoblast differentiation (Zaragosi et al., 2011; Eguchi et al., 2013; Zhang et al., 2020). In neuronal systems, members of the miR-30 family are downregulated during the differentiation of neural stem cells from pluripotent stem cells (Kulcenty et al., 2019). miR-30 is upregulated in the cortex of mice fed olive oil deprived of phenolics (Luceri et al., 2017). miR-30 is important for the conversion of fibroblasts into neurons (Soleimani et al., 2017). Moreover, miR-30 dysregulation is associated with Parkinson’s disease pathogenesis (Goh et al., 2019). Together, these observations suggest that miR-30 plays an important role in neurons. However, few studies have addressed the regulatory mechanisms of miR-30, particularly those that regulate neurite outgrowth. Here, we found that miR-30 was important for neurite outgrowth in hippocampal neurons. Overexpression of miR-30 mimics inhibited neurite outgrowth while miR-30 inhibitors promoted both the length and branching of hippocampal neurons (Figure 1), suggesting that miR-30 has an inhibitory effect on neurite outgrowth. We also identified the downstream and upstream effectors of miR-30 as spastin and MALAT1, respectively, which further elucidates how miR-30 regulates neurite outgrowth of hippocampal neurons.

MALAT1 was originally reported to be associated with cancerogenesis (Yoshimoto et al., 2016; Amodio et al., 2018; Li et al., 2018b); however, MALAT1 also plays an important role in neuronal systems (Zhang et al., 2017), where it has apoptotic and stress response functions, and in neurodegenerative diseases (Wu et al., 2013). MALAT1 sponges miR-429 to regulate the apoptosis of hippocampal neurons during hypoxic-ischemic brain damage by regulating WNT1 (Fang et al., 2019). Vitamins B1 and B12 upregulate brain-derived neurotrophic factor (BDNF) expression and its downstream PI3K/Akt signaling pathway through the MALAT1/miR-1 axis, thereby inhibiting neuronal apoptosis and reducing nerve damage in cerebral palsy rat models (Li et al., 2019). MALAT1 increases the release of inflammatory cytokines by inhibiting the ERK/MAPK signaling pathway, which upregulates neuronal apoptosis and aggravates brain damage after cerebral infarction in rats (Shi et al., 2019). Also, resveratrol improves Parkinson’s disease-like phenotype by suppressing neuronal apoptosis by modulating the MALAT1/miR-129/SNCA signaling pathway (Xia et al., 2019). In Alzheimer’s Disease models based on NGF-stimulated PC12 cells and primary cerebral cortex neurons, MALAT1 inhibits neuron apoptosis and neuroinflammation, while it stimulates neurite outgrowth *via* miR-125b-regulated PGTS2, CKD5, and FOXA1 expression (Ma et al., 2019). Here, we determined the role of MALAT1 in neurite outgrowth, but not in neuronal apoptosis. MALAT1 is known to regulate synaptogenesis by modulating gene expression networks (Bernard et al., 2010). The absence of MALAT1 can disrupt tightly controlled gene expression networks, leading to defects in signal transmission and neurite outgrowth (Cha et al., 2016). In N2a cells, MALAT1 promotes neurite outgrowth *via* the ERK/MAPK signaling pathway (Chen et al., 2016). In this study, MALAT1 was found to be important for neurite outgrowth in cultured hippocampal neurons. It upregulated the expression level of its downstream effector spastin by sponging miR-30, modulating microtubule severing and neurite outgrowth. Interestingly, MALAT1/miR-30a regulated Beclin1-dependent autophagy is involved in cell death during cerebral ischemic stroke (Guo et al., 2017) and MALAT1-sponged miR-30 targets Runx2 to promote osteoblast differentiation of adipose-derived mesenchymal stem cells (Yi et al., 2019). These data indicate that critical importance of the MALAT1/miR-30 axis, which probably targets different substrates to regulate different physiological processes.

In summary, we have characterized a regulatory axis in which MALAT1 sponges miR-30 to regulate spastin expression involved in microtubule dynamics and neuronal growth and branching. Further study of the MALAT1/miR-30/spastin axis could open a potential new avenue of research for the development of novel therapies for HSP and other neuronal dysfunctional diseases.

## Data Availability Statement

All datasets presented in this study are included in the article.

## Ethics Statement

The animal study was reviewed and approved and all animal procedures were carried out in accordance with the Guide for the Care and Use of Laboratory Animals from the NIH and were approved by the Jinan University Institutional Animal Care and Use Committee.

## Author Contributions

MT and HL conceived the project and wrote the article. TJ, ZC, and ZJ conducted all the experiments. JZ, ZL, GZ, and YL helped with biochemistry experiments and data analysis. All authors contributed to the article and approved the submitted version.

## Conflict of Interest

The authors declare that the research was conducted in the absence of any commercial or financial relationships that could be construed as a potential conflict of interest.
